# Supplementary material for: Mitofusin 1 and 2 regulation of mitochondrial DNA content is a critical determinant of glucose homeostasis
Source: Nat Commun. 2022 Apr 29;13:2340. doi: 10.1038/s41467-022-29945-7 (PMC9055072; doi:10.1038/s41467-022-29945-7)
Supplement: Supplementary file 1 — Reporting Summary [file 41467_2022_29945_MOESM1_ESM.pdf]

nature portfolio

Corresponding author(s): Scott A. Soleimanpour

Last updated by author(s): Feb 28, 2022

Reporting Summary

Nature Portfolio wishes to improve the reproducibility of the work that we publish. This form provides structure for consistency and transparency in reporting. For further information on Nature Portfolio policies, see our Editorial Policies and the Editorial Policy Checklist.

Statistics

For all statistical analyses, confirm that the following items are present in the figure legend, table legend, main text, or Methods section.

n/a	Confirmed	
☐	☒	The exact sample size (n) for each experimental group/condition, given as a discrete number and unit of measurement
☐	☒	A statement on whether measurements were taken from distinct samples or whether the same sample was measured repeatedly
☐	☒	The statistical test(s) used AND whether they are one- or two-sided<br>*Only common tests should be described solely by name; describe more complex techniques in the Methods section.*
☒	☐	A description of all covariates tested
☐	☒	A description of any assumptions or corrections, such as tests of normality and adjustment for multiple comparisons
☐	☒	A full description of the statistical parameters including central tendency (e.g. means) or other basic estimates (e.g. regression coefficient) AND variation (e.g. standard deviation) or associated estimates of uncertainty (e.g. confidence intervals)
☐	☒	For null hypothesis testing, the test statistic (e.g. F, t, r) with confidence intervals, effect sizes, degrees of freedom and P value noted<br>*Give P values as exact values whenever suitable.*
☒	☐	For Bayesian analysis, information on the choice of priors and Markov chain Monte Carlo settings
☒	☐	For hierarchical and complex designs, identification of the appropriate level for tests and full reporting of outcomes
☐	☒	Estimates of effect sizes (e.g. Cohen's d, Pearson's r), indicating how they were calculated

Our web collection on statistics for biologists contains articles on many of the points above.

Software and code

Policy information about availability of computer code

Data collection	GraphPad Prism 9 (version 9.1.0 (221))<br>FastQC (version 0.11.8)<br>STAR (version 2.6.1b)<br>RSEM (version 1.3.1)<br>DESeq2 (version 1.26.0)<br>iPathway Guide (Advaita version 1910)<br>ImageJ (version 1.53f)<br>Imaris® imaging software (version 9.5.1)<br>FlowJo (version 10.5)
Data analysis	All statistical analyses were performed using GraphPad Prism 9 (version 9.1.0 (221)).<br>High-throughput RNA sequencing data was analyzed using DESeq2 (version 1.26.0) to determine differential gene expression.<br>Pathway and GO-term analysis was performed by iPathway Guide (Advaita).<br>Immufluorescence and TEM images were analyzed on ImageJ (version 1.53f).<br>3D-renderings were generated with Imaris® imaging software (version 9.5.1).<br>Band intensities for western blots were quantified using ImageJ (version 1.53f).<br>Flow cytometry analysis was performed using FlowJo (version 10.5).

For manuscripts utilizing custom algorithms or software that are central to the research but not yet described in published literature, software must be made available to editors and reviewers. We strongly encourage code deposition in a community repository (e.g. GitHub). See the Nature Portfolio guidelines for submitting code & software for further information.

Data

Policy information about availability of data

All manuscripts must include a data availability statement. This statement should provide the following information, where applicable:

- Accession codes, unique identifiers, or web links for publicly available datasets
- A description of any restrictions on data availability
- For clinical datasets or third party data, please ensure that the statement adheres to our policy

The authors declare that data supporting the findings of this study are available within the article and its supplementary information files or from the corresponding author (S.A.S.) upon request. RNA sequencing data used for differential gene expression-, pathway- and GO- enrichment analyses have been deposited in the GEO database under the accession number GSE194204. The reference genome used for RNA sequencing was obtained from ENSEMBL [Mus_musculus - Ensembl genome browser 105]. All un-cropped western blots and data values for all figures are provided in the source data file.

Field-specific reporting

Please select the one below that is the best fit for your research. If you are not sure, read the appropriate sections before making your selection.

- [x] Life sciences
- [ ] Behavioural & social sciences
- [ ] Ecological, evolutionary & environmental sciences

For a reference copy of the document with all sections, see nature.com/documents/nr-reporting-summary-flat.pdf

Life sciences study design

All studies must disclose on these points even when the disclosure is negative.

Sample size	Sample-size calculation was performed based on the integration of several factors, including estimates of necessary sample sizes from our >20 years experience in the field, power calculation guidance per G*Power, and were subject to the availability of genetically-modfied mice from complex breeding schemes. Statistical methods were chosen prior to completion of all experiments. Equivalent representation of both sexes was similar in all sample groups.
Data exclusions	No data were excluded from the analyses
Replication	All experimental findings were replicated successfully in pancreatic islets obtained from multiple age-matched mice. All experiments and replications were performed independently by multiple technical operators with successful and consistent results between all operators.
Randomization	The genotype of all mice was known prior to allocation into experimental groups. Animals of suitable genotypes were then randomly allocated into study groups for the numerous experimental endpoints completed within this study.
Blinding	The authors were blinded to the genotype of experimental mice while performing all studies. RNA sequencing was performed by the Bioinformatics Core at University of Michigan, who were blinded to the genotype of RNA samples submitted during initial processing of results. RNA sequencing data was then processed by unbiased bioinformatics pipelines prior to evaluation by the authors.<br>Authors were then unblinded upon the completion of studies for downstream data assessment. Data were also assessed by multiple operators to prevent bias.

Reporting for specific materials, systems and methods

We require information from authors about some types of materials, experimental systems and methods used in many studies. Here, indicate whether each material, system or method listed is relevant to your study. If you are not sure if a list item applies to your research, read the appropriate section before selecting a response.

Materials & experimental systems

n/a	Involved in the study	
[ ]	[x]	Antibodies
[x]	[ ]	Eukaryotic cell lines
[x]	[ ]	Palaeontology and archaeology
[ ]	[x]	Animals and other organisms
[x]	[ ]	Human research participants
[x]	[ ]	Clinical data
[x]	[ ]	Dual use research of concern

Methods

n/a	Involved in the study	
[x]	[ ]	ChIP-seq
[ ]	[x]	Flow cytometry
[x]	[ ]	MRI-based neuroimaging

Antibodies

Antibodies used	[Antibody/ Dilution used/ Manufacturer and Catalog number]

	Cyclophilin B/ 1:5000/ ThermoFisher PA1-027A<br>Anti-DNA/ 1:50/ American Research Products (ARP) 03-61014<br>Drp1/ 1:1000/ Cell Signaling Technology 8570<br>Glucagon/ 1:2000/ SantaCruz sc-13091<br>Insulin/ 1:250/ Dako A0564<br>Insulin/ 1:100/ Abcam ab7842<br>LonP1/ 1:1000/ Proteintech 15440-1-AP<br>Mfn1/ 1:1000/ Abcam ab126575<br>Mfn2/ 1:1000/ Abcam ab56889<br>mt-Cytb/ 1:500/ ProteinTech 55090-1-AP<br>Opa1/ 1:1000/ Transduction laboratories 612606<br>Total OXPHOS antibody cocktail/ 1:1000/ Abcam ab110413<br>Pdx1/ 1:250/ Abcam ab47383<br>Polg/ 1:1000/ Abcam ab128899<br>SDHA/ 1:100/ Abcam ab14715<br>Somatostatin/ 1:500/ Abcam ab30788<br>Ssbp1/ 1:250/ Atlas antibodies HPA002866<br>Anti-human TFAM/ 1:1000/ PhosphoSolutions 1999-hTFAM<br>Anti-mouse Tfam/ 1:1000/ PhosphoSolutions 2001-TFAM<br>Tom20/ 1:1000/ Cell Signaling Technology 42406<br>Twnk/ 1:1000/ Proteintech 13435-1-AP<br>Vinculin/ 1:5000/ Millipore CP74
Validation	Specificity of Mfn1 (Abcam ab126575) and Mfn2 (Abcam ab56889) to their respective target protein was confirmed by western blot using samples obtained from Mfn1-/- and Mfn2-/- mice.<br>All other antibodies were validated by the supplier and by references in the literature provided by the supplier for immunohistochemistry and western blot applications towards their respective mouse or human immunogen.

Animals and other organisms

Policy information about studies involving animals; ARRIVE guidelines recommended for reporting animal research

Laboratory animals	Mfn1-loxP/loxP and Mfn2-loxP/loxP: [Species: Mus musculus, Strain: C57BL/6N, Gender: male and female, Age: 6-15 weeks old]<br>db/+ and db/db mice: [Species: Mus musculus; Strain: BKS; Gender: male and female; Age: 10-12 weeks old]
Wild animals	The study did not involve wild animals.
Field-collected samples	The study did not involve field-collected samples.
Ethics oversight	All animal protocols have been approved by the Institutional animal care and use committee (IACUC) at University of Michigan

Note that full information on the approval of the study protocol must also be provided in the manuscript.

Flow Cytometry

Plots

Confirm that:

- [x] The axis labels state the marker and fluorochrome used (e.g. CD4-FITC).
- [x] The axis scales are clearly visible. Include numbers along axes only for bottom left plot of group (a 'group' is an analysis of identical markers).
- [x] All plots are contour plots with outliers or pseudocolor plots.
- [x] A numerical value for number of cells or percentage (with statistics) is provided.

Methodology

Sample preparation	Pancreatic islets were stained with MtPhagy dye for 3 hours and then dispersed into single cells. Single cells were then stained with DAPI and Fluozin-3 for 30 minutes prior to resuspension in phenol red-free islet culture medium
Instrument	LSR Fortessa flow cytometer (BD Biosciences)
Software	FlowJo (Tree Star Inc.)
Cell population abundance	Flow cytometry in figure S4E was used to measure median fluorescence intensity under the specified excitation/emission channels. No FACS sorting was performed.
Gating strategy	Cell population were identified based on FSC/SSC morphological parameters to only include single dispersed islet cells. Live-cells were gated using DAPI staining. Beta-cells were identified by Fluozin-3 staining. Figure exemplifying the gating strategy is

provided in the supplementary information of this manuscript as Figure S8.

☒ Tick this box to confirm that a figure exemplifying the gating strategy is provided in the Supplementary Information.
